# Exposure to low intensity ultrasound removes paclitaxel cytotoxicity in breast and ovarian cancer cells

**DOI:** 10.1186/s12885-021-08722-7

**Published:** 2021-09-01

**Authors:** Celina Amaya, Shihua Luo, Julio Baigorri, Rogelio Baucells, Elizabeth R. Smith, Xiang-Xi Xu

**Affiliations:** 1 Department of Radiation Oncology, Sylvester Comprehensive Cancer Center, University of Miami Miller School of Medicine, Papanicolaou Building, Room 415 [M877], 1550 NW 10th Avenue, Miami, FL 33136 USA; 2 HHMI High School Scholars Program, Department of Undergraduate Research and Community Outreach, University of Miami, Miami, FL 33146 USA; 3 Department of Cell Biology, University of Miami Miller School of Medicine, Miami, FL 33136 USA

**Keywords:** Ultrasound wave, Microtubule, Taxol/paclitaxel, Cytotoxicity, Lysosomal degradation, Ovarian cancer, Breast cancer

## Abstract

**Background:**

Paclitaxel (Taxol) is a microtubule-stabilizing drug used to treat several solid tumors, including ovarian, breast, non-small cell lung, and pancreatic cancers. The current treatment of ovarian cancer is chemotherapy using paclitaxel in combination with carboplatin as a frontline agent, and paclitaxel is also used in salvage treatment as a second line drug with a dose intensive regimen following recurrence. More recently, a dose dense approach for paclitaxel has been used to treat metastatic breast cancer with success.

Paclitaxel binds to beta tubulin with high affinity and stabilizes microtubule bundles. As a consequence of targeting microtubules, paclitaxel kills cancer cells through inhibition of mitosis, causing mitotic catastrophes, and by additional, not yet well defined non-mitotic mechanism(s).

**Results:**

In exploring methods to modulate activity of paclitaxel in causing cancer cell death, we unexpectedly found that a brief exposure of paclitaxel-treated cells in culture to low intensity ultrasound waves prevented the paclitaxel-induced cytotoxicity and death of the cancer cells. The treatment with ultrasound shock waves was found to transiently disrupt the microtubule cytoskeleton and to eliminate paclitaxel-induced rigid microtubule bundles. When cellular microtubules were labelled with a fluorescent paclitaxel analog, exposure to ultrasound waves led to the disassembly of the labeled microtubules and localization of the signals to perinuclear compartments, which were determined to be lysosomes.

**Conclusions:**

We suggest that ultrasound disrupts the paclitaxel-induced rigid microtubule cytoskeleton, generating paclitaxel bound fragments that undergo degradation. A new microtubule network forms from tubulins that are not bound by paclitaxel. Hence, ultrasound shock waves are able to abolish paclitaxel impact on microtubules.

Thus, our results demonstrate that a brief exposure to low intensity ultrasound can reduce and/or eliminate cytotoxicity associated with paclitaxel treatment of cancer cells in cultures.

**Supplementary Information:**

The online version contains supplementary material available at 10.1186/s12885-021-08722-7.

## Introduction

Paclitaxel (Taxol®) is an important component of the chemotherapeutic regime for several solid tumors, including those of lung, breast, prostate, ovary, and Kaposi’s sarcomas [1–5]. Paclitaxel was first developed and approved for use in treating ovarian carcinomas [5, 6], and it is still a key drug in the current management of ovarian cancer, used as a frontline agent in combination with platinum-containing drugs, such as carboplatin and cisplatin [7], and also in salvage treatment as a second line drug with a dose intensive regimen following recurrence [8–11]. Paclitaxel generally is highly effective with tolerable side effects, but sensory peripheral neuropathy, presenting as numbness and pain of feet and hands, is often the dose-limiting toxicity of the agent [12–15]. Although attempts have been explored to reduce or prevent damage to the extremities, such as hypothermia cooling gloves and socks [16, 17], currently no effective or satisfactory methods or drugs are available to reverse this daunting side effect [18, 19]. Similarly, alopecia (hair loss) is another detrimental side effect of paclitaxel chemotherapy [20–22].

Paclitaxel binds tightly to the beta-tubulin protein within microtubules, and stabilizes the microtubule filaments [2, 23, 24]. This stabilization of microtubules interferes with microtubule dynamics and leads to growth arrest and subsequently apoptosis [2, 25, 26], which is thought to underlie the anti-cancer mechanism of action. Paclitaxel has also been shown to induce aberrant multipolar division in mitosis, leading to chromosome mis-segregation and subsequently apoptotic cell death [27, 28]. Because it targets microtubule function in mitosis, paclitaxel is generally considered a microtubule stabilizing and anti-mitotic cancer drug [2, 29]. However, non-mitotic targeting of paclitaxel mechanisms are also suggested [30–33]. Factors related to microtubule dynamic stability appear to underlie the sensitivity and resistance of cancer cells to paclitaxel [1, 34, 35].

Ultrasound technologies have extensive applications in medicine, either for diagnosis (sonogram) or therapy [36–38]. Typically, ultrasound with extremely low intensity (1–50 mW/cm^2^) and high frequency (3–5 MHz) is used for diagnostic (imaging) purposes. High intensity (> 8 W/cm^2^, 20–60 kHz) ultrasound that can deliver strong energy is used for surgery and disruption through heating and acoustic cavitation. The medical application of ultrasound with an intensity that is low yet sufficiently high to produce biological activity is known as ultrasound physiotherapy [38], which uses a sufficiently strong but non-disruptive ultrasound shock waves (0.5–3.0 W/cm^2^) [38–40]. The most commonly used devices produce ultrasound waves with frequencies either around 1 MHz or 20–150 kHz (known as long wavelength ultrasound). Several studies report similar effects by either 1–3 MHz or 45 kHz ultrasound waves on cells and tissues [41, 42]. The majority of ultrasound in medical application of physiotherapy is using the frequency in the range of 1–3 MHz, which traditionally is thought to produce less cavitation and thus tissue damage. However, more recent laboratory findings indicate that the low frequency (20 to 100 KHz) ultrasound seems to produce a stronger biological impact [42–44], and at the same time seems to produce no cell and tissue damage [45–47]. With the development of better low frequency ultrasound devices [48], the use of low frequency ultrasound for medical procedure gains interests [49], improving cardia function [46, 50], and treating neuropathy-induced ulcers [51]. The new development in ultrasound physiotherapy provides rationale to study the impact of low frequency ultrasound waves on cells.

Many of the studies attribute the physiological activities of ultrasound waves to heating; however, non-heating effects, such as changes in gene expression, mechanical transduction, and release of cytokines, have also been noted [43, 44, 52]. In particular, ultrasound disrupts the cellular cytoskeleton [53–55]. The force transduced by ultrasound can transiently disrupt actin and microtubule cytoskeletons, which then reassemble without significant impact on cell structure and survival [53–56]. Studies also found that low intensity ultrasound caused chromosomal damage and DNA breaks [57]. Focused, low intensity ultrasound may also be useful for targeting tumors [58].

Our initial experimental rationale was to test if low intensity ultrasound could cause DNA damage and synergize with paclitaxel, replacing the use of a platinum agent in chemotherapy. Unexpectedly, however, we found that ultrasound treatment can effectively and completely neutralize the cytotoxic effect of paclitaxel on cells in culture. Based on the results, we conclude that a brief exposure of low intensity ultrasound can reverse paclitaxel-induced cytotoxicity in cancer cells, and provide a possible mechanism.

## Methods

### Reagents

Taxol/paclitaxel were purchased from Sigma-Aldrich, Inc., and the dry powder was prepared for 100 μM stock solutions in DMSO. Tissue culture flasks (trade mark Falcon), tissue culture media, trypsin, and 100X antibiotic-antimycotic solution (Cellgro, Mediatech, Inc) were purchased from VWR Inc. (Springfield, NJ). Alexa Fluor 488 and 555 conjugated secondary antibodies were purchased from Life Sciences Inc. (Eugene, Oregon) for use in immunofluorescence microscopy. Primary antibodies: anti-Lamin A (1:400, H-102, rabbit polyclonal IgG); mouse monoclonal anti-Lamin B (1:300, sc373918); and goat polyclonal anti-Lamin B (1:400, sc6216) were from Santa Cruz Biotechnology Inc.; rabbit polyclonal anti-Lamin B1 (1:1000, ab16048) and mouse monoclonal anti- αTubulin (1:500, 66,031) were from Abcam and Proteintech, respectively. Mouse monoclonal anti-alpha-tubulin, were purchased from Sigma-Aldrich, Inc. (St. Louis, MO). More details of the reagents used are also reported previously [59–62].

### *Cell culture*

Ovarian cancer cells including MCF 7, MDA-MB-231, A2780, purchased from ATCC (American Tissue Culture Collection), and OVCAR3, − 4, − 5, − 8, and − 10 originally obtained from Hamilton lab [59, 60] were used in the experiments. These cells were cultured in RPMI-1640 media supplemented with 10% fetal bovine serum and 1X penicillin/streptomycin, as described previously [59, 61, 62]. For serum-free experiments, cells were cultured in RPMI with 1% BSA and 1X penicillin/streptomycin. All cells were maintained at 37 °C in a humidified atmosphere of 5% CO_2_.

#### Ultrasound exposure

**Fig. 1:**
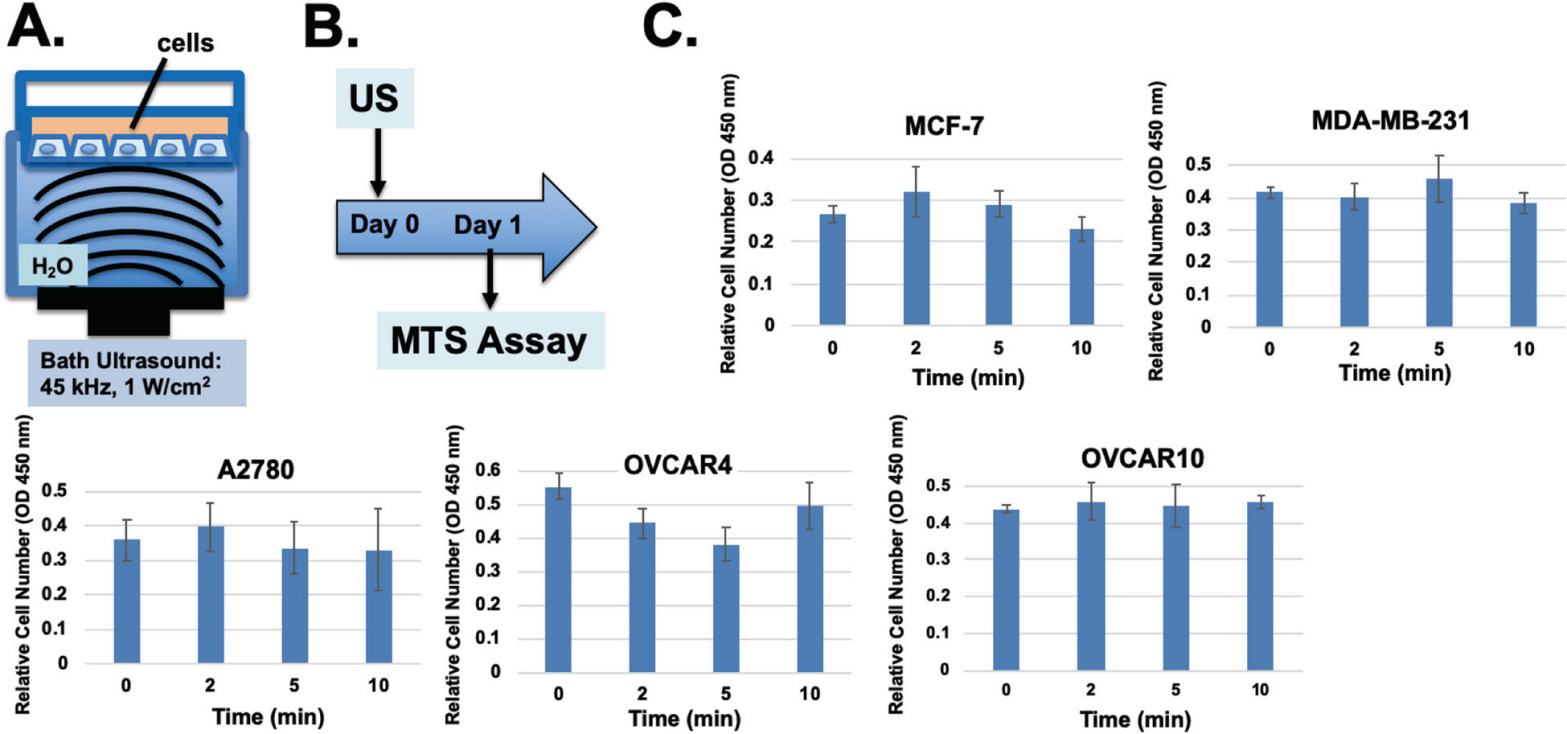
Ultrasound treatment of cultured cells had no significant impacts on cell growth and survival. **A** Illustration of a devise for the transmission of ultrasound wave through water to monolayer cells on tissue culture dish. The ultrasound used was 45 KHz, 1 W/cm^2^. **B** The experimental protocol is illustrated: cells were exposed to ultrasound on day1 and cell numbers were assayed on day 2. **C** Cell numbers were determined using WST-1 proliferation assay kit assay of cells exposed to 0, 2, 5, and 10 min of ultrasound

We have acquired and tested both the bath and probe types of ultrasound devices for our study (Fig. 1a). The bath device produces 45 or 150 kHz, 1–3 W/cm^2^ ultrasound waves with adjustable frequency and energy levels (Crest PowerSonic P1100 Model, US Ultrasonics. LLC, Richmond VA USA).

Cells were plated on 6-well tissue culture plates, and cultured with 0.5 ml of medium. For treatment with ultrasound, the tissue culture plate was placed on the surface of the water bath with a temperature of the bath set at 35 °C. Using a costumed-make manifold that holds the dish and fitted on the ultrasound bath, the tissue culture plate was immersed in the bath with the bottom of the plate around 2–3 mm under water surface. Generally, the power level was set at 1 W/cm^2^. Immediately following exposure to ultrasound, the plate was placed back into cell culture incubator.

#### Cell proliferation assay

Cell numbers were determined using an WST-1 cell proliferation kit from Roche. Briefly, at the determined time points, the cell culture medium was replaced with freshly prepared reaction buffer composed of DMEM/F12 media containing 10% WST-1 solution and incubated at 37 °C for 1 h. The absorbance at 450 nm of the media measures the accumulation of the soluble formazan dye produced by viable cells, which correlates with relative cell number of the same cell types.

#### Immunofluorescence microscopy

The procedure has been described previously [62–64]. Briefly for immunofluorescence microscopy, adhered cells on glass coverslips were washed twice with PBS at room temperature, fixed with 4% paraformaldehyde for 15 min, and permeabilized with 0.1% Triton X-100 for 5 min. Then, the cells were washed three times with PBS, blocked with 5% BSA in PBS containing 0.1% Tween-20 for 1 h, and incubated overnight at 4 °C with primary antibodies in 5% BSA in PBS. AlexaFluor 488-conjugated (green fluorescence) or AlexaFluor 555-conjugated (red fluorescence) secondary antibodies were used. Cells were washed three times, counterstained with DAPI, then mounted and sealed in ProLong Gold antifade reagent (Thermo Fisher Inc). Immunofluorescence was viewed using a 100X objective lens on Zeiss Axio Observer Z1 using AxioVision software.

## Results

### A brief exposure to low intensity ultrasound does not affect cancer cell survival and growth in culture

We performed initial testing of ultrasound of both 1 MHz and 45 kHz on modulating paclitaxel treatment of cancer cells in cultures. Although either ultrasound frequency produced similar effects on cell survival, the long wavelength ultrasound appeared to give a stronger and consistent impact with similar intensity, consistent with that reported [42]. Thus, we chose to focus on our first study using the 45 kHz ultrasound set up.

First, we examined the effect of low frequency low intensity ultrasound exposure on survival of monolayer cells in tissue culture dishes with a water bath ultrasound set up (Fig. 1a). Several lines of breast (MCF-7, MDA-MB-231) and ovarian (A2780, OVCAR3, OVCAR4, OVCAR5, OVCAR10) cancer cells were plated as monolayers in 6-well culture dishes at around 70% density with 2 ml of medium per well covering the cells. The tissue culture plate was hold with a manifold that submerged the plate with the bottom at a level approximately 2 to 3 mm beneath the water surface of the bath sonicator. Here the temperature of the water bath was set to 35 °C to prevent potentially heating over 37 °C, and the ultrasound frequency was set at 45 kHz, with an energy output of 1 W/cm^2^. Following a brief ultrasound exposure, the tissue culture dishes were placed back into tissue culture incubator and the cell number was determined by WST-1 proliferation assay 1 day after ultrasound exposure (Fig. 1b).

Exposure of cells to 45 kHz ultrasound for 2 to10 min had no major effect on cell survival and proliferation measured 1 day after exposure in all cell line tested (Fig. 1c). The ultrasound treatment did not alter the temperature of the culture medium. Additional experiments also confirmed that cell numbers were not affected 2 day after ultrasound treatment (not shown). In some lines (A2780), a fraction (around 10%) of cells appeared to detach from the culture dish after a 10-min exposure to ultrasound immediately after treatment; however, the treatment had no significant impact on cell survival and growth. Presumably, the cells re-attached and continue to grow. Thus, a brief exposure of ultrasound alone did not affect cells in culture. However, it was noted that prolong exposure to ultrasound such as 15 min or longer resulted in increased detachment of cells from the culture plate and progressively loss of cell viability. Especially, higher ultrasound intensity (such as 3 W/cm^2^) with longer exposure also significantly reduced cell survival.

### A brief exposure to low intensity ultrasound eliminates paclitaxel cytotoxicity in breast and ovarian cancer cells in cultures

**Fig. 2:**
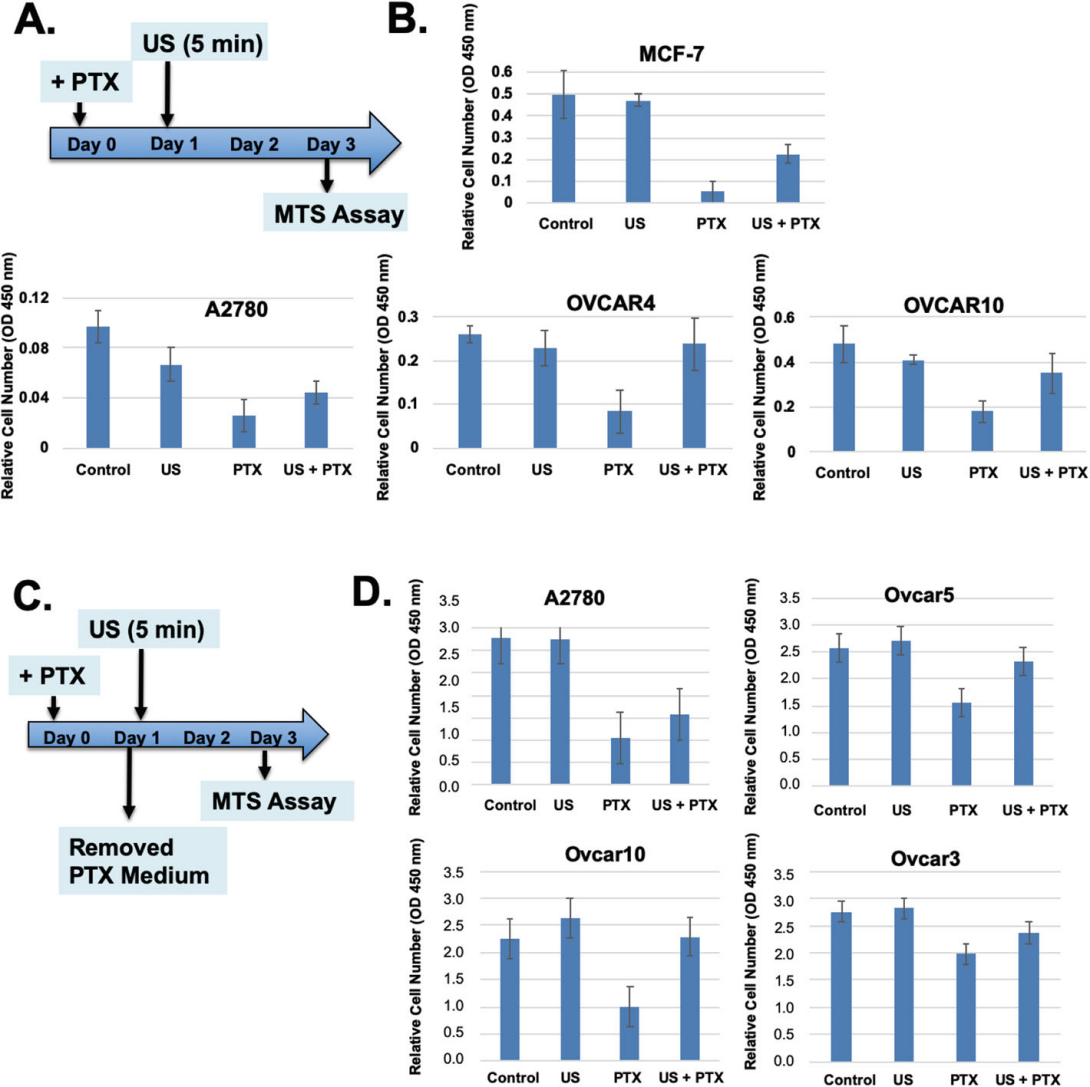
Paclitaxel mediated cell killing and reversal by ultrasound in the absence of continuous exposure to extracellular paclitaxel. **A** The experimental protocol is illustrated. **B** Ovarian cancer cells were treated with or without paclitaxel (1 nM, + PTX), and in next day, the cells were exposed to ultrasound for 5 min. In 48 h, the cell number were determined by WST-1 assay with triplicate samples. The error bars represent standard deviations. The differences in cell numbers between PTX and PTX + US are statistically significant determined by Student’s T-test. The *p* values are 0.009, 0.007, and 0.015 for MCF-7, OVCAR4, and OVCAR10 respectively. **C** The experimental protocol is illustrated. The protocol is similar to those presented above. However, paclitaxel in the medium was removed before ultrasound exposure at Day 1. **D** Breast and ovarian cancer cells were treated with or without paclitaxel (1 nM, + PTX), and in about 24 h, the medium was changed and the cells were exposed to ultrasound (+ US, 45 KHz, 1 W/cm2) for 5 min. At day 3, the cell numbers were determined by WST-1 assay with triplicate samples. The differences in cell numbers between PTX and PTX + US are statistically significant with p value of 0.07, 0.02, 0.007, and 0.07 for A2780, OVCAR5, OVCAR10, and OVCAR3 respectively, determined by Student’s T-test

In an initial experiment with an intend to determine the potential synergistic effect of paclitaxel and low intensity ultrasound in killing ovarian cancer cells, we observed a surprising result that actually ultrasound reversed the cytotoxicity of paclitaxel (Fig. 2). The concerntrations of paclitaxel used in studying cancer cells in cultures are generally to be in a range between 0.1 to 100 nM, and we have recently determined the the dosage for 50% cell killing was around 0.3 nM [64]. Thus, we chose a concerntration of 1 nM paclitaxel to be used in the current experimental settings. In the experiments, paclitaxel was added to cells in culture for a total of 3 days, and a 5-min ultrasound treatment was performed on day 1, following 2 days in culture before determination of cell number (Fig. 2a). In the absence of paclitaxel, ultrasound exposure had little effect on cell number by the end of day 3. As expected, paclitaxel treatment of the ovarian and breast cancer cells by severely reduced cell numbers to approximately 10–25% of control (Fig. 2b). However, the cell numbers measured at the end of day 3 were higher in all lines when the paclitaxel treated cells were exposed to ultrasound for 5 min on day 1 (Fig. 2b). Surprisingly, ultrasound treatment appeared to reduce or remove the inhibitory effect of paclitaxel.

We also performed the experiments in a slightly different protocol; that medium was changed at day 1 to fresh medium without paclitaxel, prior to ultrasound exposure (Fig. 2c). Replacing paclitaxel containing medium to fresh medium without paclitaxel after 1 day had little changes in the cytotoxicity of paclitaxel observed at day 3 (Fig. 2d), indicating the impact of paclitaxel after incubation for 1 day lingered to day 3. This phenomenon is previously known, that a short-term exposure of cancer cells to paclitaxel produced persistent growth inhibitory activity [65]. Ultrasound exposure for 5 min was also able to reverse cytotoxicity from the one-day exposure of paclitaxel, suggesting the impact of ultrasound was to remove the lingering activity of paclitaxel for cell killing on day 2 and 3 (Fig. 2d).

Thus, the results indicate that paclitaxel exposure for 24 h is sufficient to kill cells over the next 2 days without the need for paclitaxel in the medium, and ultrasound exposure reduces the effect of paclitaxel activity retained in the cells from the initial exposure.

### Reversal of paclitaxel cytotoxicity depends on timing and schedule of ultrasound exposure

**Fig. 3:**
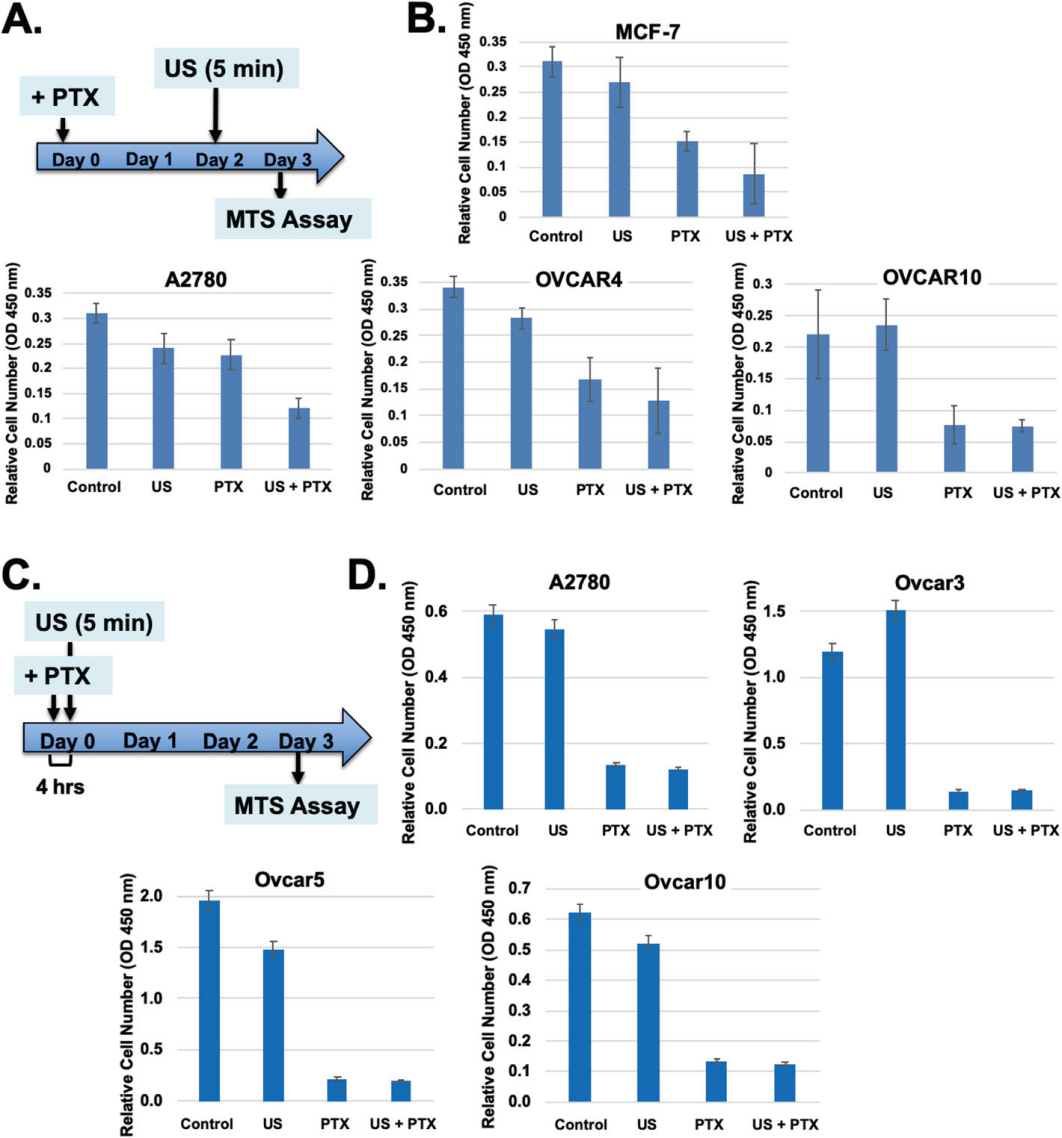
Paclitaxel mediated cell killing and reversal by ultrasound depends on schedule and timing. **A** The experimental protocol is illustrated. Ultrasound was applied to the cells at Day 2 of paclitaxel treatment. **B** Breast and ovarian cancer cells (MCF-7, A2780, OVCAR4, OVCAR10) were treated with or without paclitaxel and ultrasound, and cell number was determined on Day 3. **C** The experimental protocol is illustrated: ultrasound was applied to the cells 4 h after paclitaxel treatment. **D** Ovarian cancer cells (A2780, OVCAR33, OVCAR5, OVCAR10) were treated with or without paclitaxel and ultrasound, and cell number was determined on Day 3. The standard deviations are presented as error bars. The differences in cell numbers between PTX and PTX + US are statistically not significant (*P* > 0.07) determined by Student-T test, for all cell lines tested

The observation that ultrasound treatment reduced paclitaxel cytotoxicity was rigorously repeated and determined to be robust, though sensitive to schedules and timing of ultrasound exposure. We designed experiments with various schedules of ultrasound exposure to determine the optimal time to reverse paclitaxel cytotoxicity and to understand mechanisms (Fig. 3). In one set of experiments when ultrasound was given 4 h after paclitaxel and over a 3-day time course (Fig. 3a), there was no difference on cell numbers compared to paclitaxel alone, indicating no rescue (Fig. 3b). When delivered on day 2, ultrasound also did not alter paclitaxel cytotoxicity (Fig. 3c,d). Instead, the optimal time to administer ultrasound to reverse paclitaxel inhibition of cell proliferation was found to be 8 to 24 h after paclitaxel was added. The experiments were repeated and performed in multiple cell lines, with results supporting that reversal of paclitaxel cytotoxicity by a brief exposure to ultrasound is reproducible, although the dosage of paclitaxel and the timing of ultrasound exposure influence the degree of activity.

### Ultrasound shock wave causes transitory disruption of the microtubule cytoskeleton

**Fig. 4:**
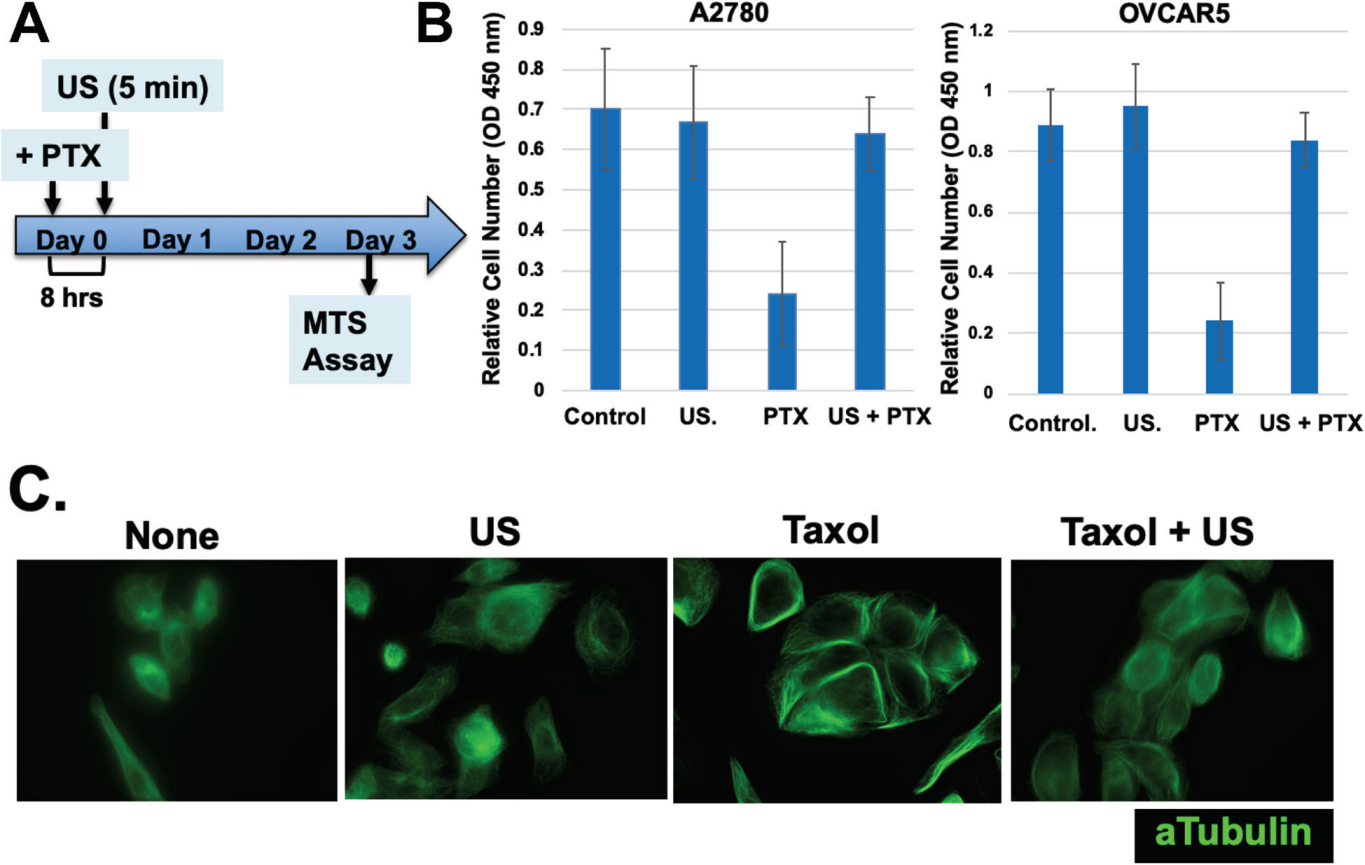
Correlation between reversion of cell growth and remodeling of microtubule cytoskeleton. **A** Ovarian cancer cells (OVCAR5, OVCAR4, OVCAR8, OVCAR10, and A2780) were treated with or without paclitaxel (1 nM, Taxol), and in 8 h, the cells on tissue culture dish were exposed to ultrasound (+ US, 40 KHz, 2.5 W/square inch) for 5 min. **B** Cell number was determined by WST-1 assay on Day 3. The differences in cell numbers between PTX and PTX + US are statistically significant determined by Student-T test: *p* = 0.01 and 0.008 for A2780 and OVCAR5 respectively. **C** After recovery from ultrasound exposure for 2 h, the cells were analyzed by immunofluorescence microscopy for alpha tubulins. A representative example of OVCAR5 cells is shown

Low intensity ultrasound is known to disrupt the microtubule cell cytoskeleton [53–56]. Indeed, we confirmed that a brief exposure of ultrasound disrupted microtubules, although the structure reformed within hours. An experiment is shown in Fig. 4a, in which cells were treated with paclitaxel for 8 h, then were exposed to ultrasound for 5 min, and cell number was determined in day 3. Here, paclitaxel treatment greatly reduced the cell number determined after 3 days (Fig. 4b), though an exposure to ultrasound nearly completely prevented cell death or suppression of growth (Fig. 4b). Paclitaxel treatment resulted in the intense immunostaining of microtubule filaments, which was abolished by low intensity ultrasound (Fig. 4c). Here, the staining of microtubules was performed 2 h after ultrasound exposure, when the microtubule cytoskeleton had reformed. The microtubule cytoskeleton in paclitaxel and then ultrasound treated cells appeared to share the same morphology as control (no paclitaxel) cells, even though paclitaxel was not removed from the medium (Fig. 4c). Thus, we were able to use setting of ultrasound with nearly no impact on cell viability and growth but with complete reversion of paclitaxel cytotoxicity (Fig. 4).

**Fig. 5:**
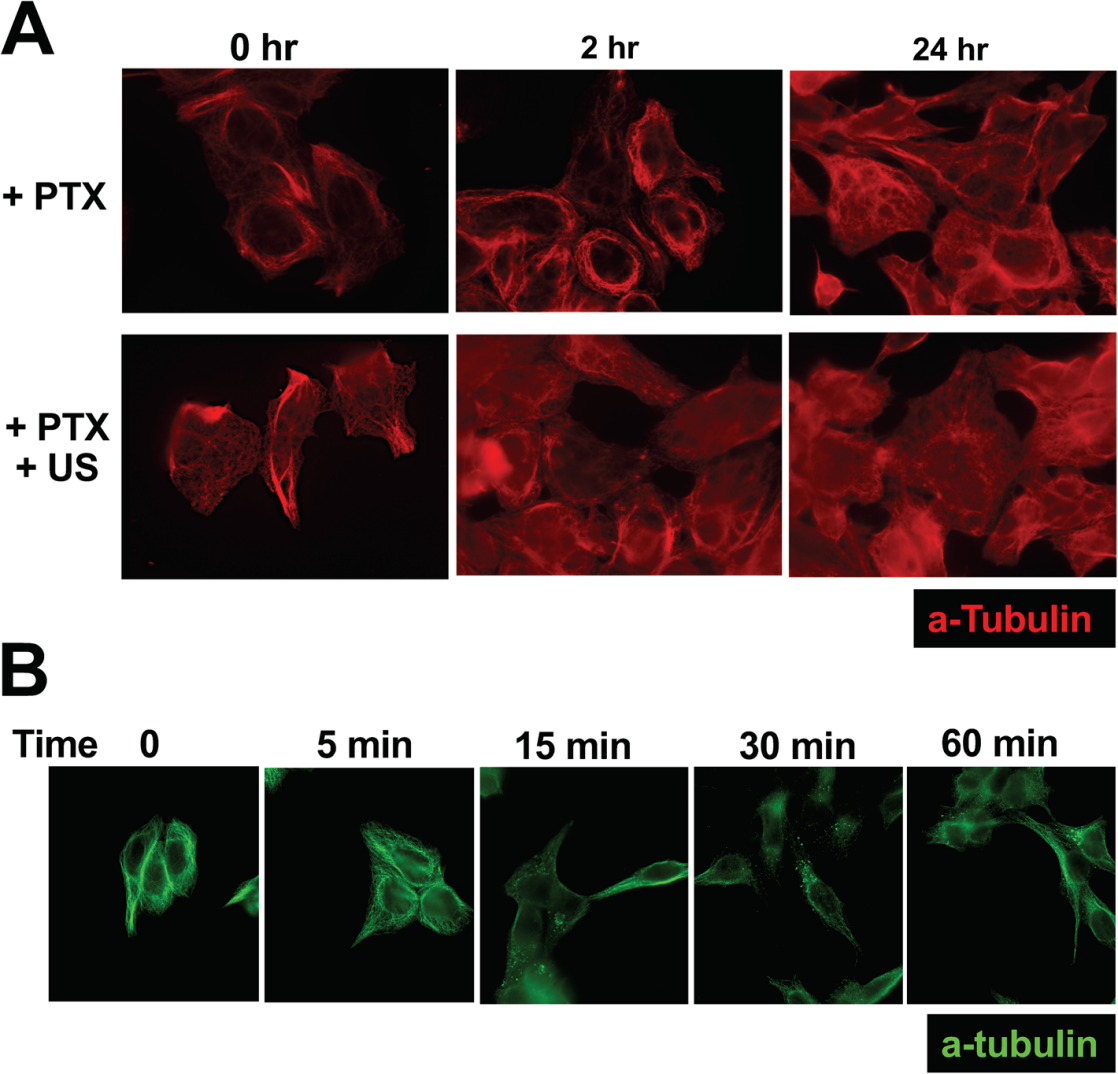
Impacts of paclitaxel and ultrasound on microtubule filaments. **A** Breast and ovarian cancer cells (MCF-7, OVCAR3,OVCAR5, OVCAR4, OVCAR8, OVCAR10, and A2780) were treated with paclitaxel (1 nM, Taxol), and in 8 h, the cells on tissue culture dish were exposed to ultrasound (+ US, 40 KHz, 1 W/cm^2^) for 5 min. Following ultrasound treatment, at 0.2, and 24 h, the cells were fixed and processed for immunostaining for alpha-tubulin. An example of OVCAR5 cells is shown. **B** Cells were treated with paclitaxel (1 nM, Taxol), and in 8 h, the cells on tissue culture dish were exposed to ultrasound (+ US, 40 KHz, 1 W/cm^2^) for 5 min. Following ultrasound treatment, at 0, 5, 15, 30 and 60 min, the cells were fixed and processed for analyses by immunofluorescence microscopy for alpha tubulins. A representative example of A2780 cells is shown

We also observed that paclitaxel-induced microtubule bundling persisted over 48 h (Fig. 5a). In contrast, ultrasound treatment eliminated paclitaxel-induced microtubule bundles in the cells over the next 24 h (Fig. 5a).

A careful examination of the microtubule network immediately and over time after ultrasound treatment showed that ultrasound only subtly altered microtubules as detected by alpha-tubulin immunostaining (Fig. 5b). The staining appeared slightly more diffuse immediately (5 min) after ultrasound treatment (Fig. 5b), although microtubule bundles were still present. Subsequently, from 15 to 60 min after ultrasound of the cells, alpha-tubulin-stained spots appeared (Fig. 5b). The spots then faded away in next 1–2 h (such as seen in Fig. 4b).

### Lysosomal degradation of paclitaxel analogs labeled microtubules following exposure to ultrasound shock wave

**Fig. 6:**
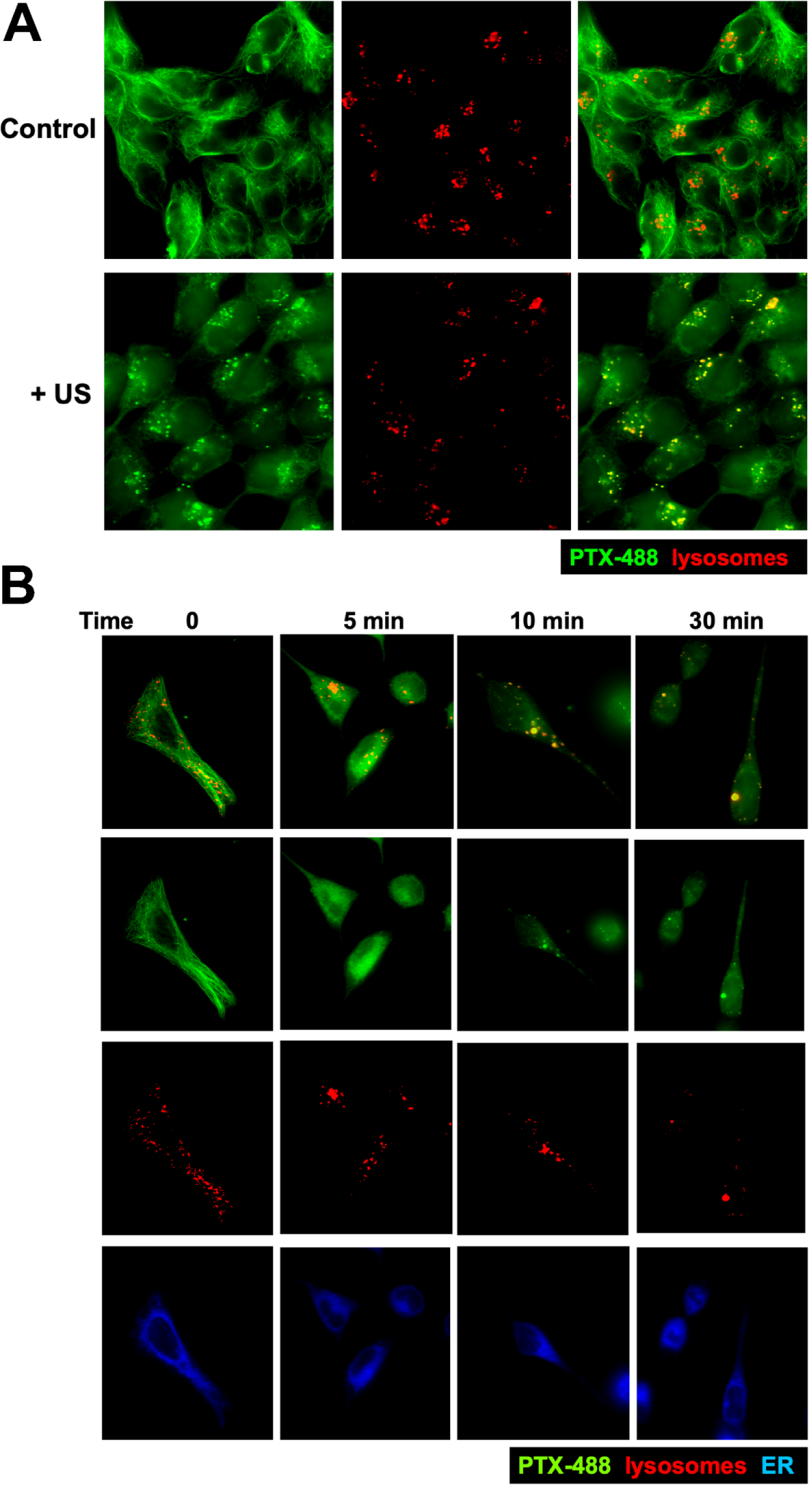
Re-localization of fluorescence labeled Taxol to lysosomes following ultrasound. **A** Breast and ovarian cancer cells were added with fluorescence labeled Taxol analog (488-PTX) (1 nM) and Lyso-marker for 60 min. The cells on tissue culture dish were exposed to ultrasound (+ US, 40 KHz, 1.0 W/cm^2^) for 5 min. Representative 488-PTX images were taken before (control) and after exposure to ultrasound. The signals from 488-PTX and Lyso-Marker were combined/overlaid. An example of A2780 cells is shown. **B** Cells incubated with fluorescence labeled Taxol analog (488-PTX) (1 nM), ER-Tracker™, and Lysomarker (BioTracker LYSO-TP Live Cell Dye) markers for 60 min before exposure to ultrasound for 5 min. After exposure to ultrasound at time 0 (no ultrasound), 5, 10, 30 min, the cells were imaged. Representative images of OVCAR5 cells are shown

We examined the effects of ultrasound on the microtubule cytoskeleton by immunofluorescence microscopy using a fluorescence-labeled paclitaxel analog (488-PTX) (Oregon Green™ 488 Taxol, from ThermoFisher). Within 30 min after 488-PTX was added to cultures, the fluorescence signals uniformly labeled the microtubule cytoskeleton (Fig. 6a, control; Fig. 6b, 0 time). Without further perturbation, the fluorescence labels on cellular microtubules persisted, at the least for 24 h. We found that a brief ultrasound treatment disrupted irreversibly the paclitaxel-labeled microtubule network. The fluorescence signals initially became diffuse, then the fluorescent molecules (presumably paclitaxel-bound tubulin or microtubule fragments) subsequently formed spots, which were identified as lysosomes using the lysosome marker, BioTracker LYSO-TP Live Cell Dye (from Millipore/Sigma) (Fig. 6).

Comparing to alpha-tubulin immunostaining staining, it appeared that nearly all the fluorescence Taxol-labeled microtubules were irreversibly disrupted by a brief exposure to ultrasound (Fig. 6a,b). In 30 to 60 min following ultrasound treatment, essentially all the fluorescence signals fated, while the labels on untreated cells retained. Thus, the elimination of paclitaxel bound microtubules seemed to correlate with the reversal of paclitaxel cytotoxicity.

The analyses of low intensity ultrasound in disrupting microtubules and reduction of paclitaxel cytotoxicity in cultured cells were first observed 3 years ago, and the lab has continued the investigation over the years in multiple cell lines. Only representative results are shown here, and a few results are included as supplementary figures. The impact of the low intensity ultrasound appears to be very robust and reproducible. Although subtle differences in timing. Degree of responses, and behaviors between different cell lines were observed, the general conclusion that low intensity ultrasound disrupt microtubules and reduce/eliminate paclitaxel cytotoxicity is applicable to all lines.

## Discussion

In this study, we have made a surprising discovery that ultrasound treatment can effectively and nearly completely neutralize the cytotoxic effect of paclitaxel on cells in culture. Thus, we have discovered a technology and procedure in locally reversing paclitaxel cytotoxicity.

Our results confirm that low intensity ultrasound transiently disrupts the microtubule cytoskeleton, which can reform rapidly [53–56]. However, we speculate that ultrasound waves break the rigid, paclitaxel-bound microtubules into small filaments or individual tubulins that are still bound to paclitaxel. The unbound tubulin monomers likely form a new cytoskeleton during the recovery from ultrasound disruption. The paclitaxel-bound tubulins, either as fragments of broken microtubule filaments or as tubulin heterodimers/monomers, relocate to lysosomes for degradation.

Ultrasound physiological therapies have been widely used in various treatments of medical conditions including pain relief, wound healing, etc. [38–40]. Anecdotal accounts support many of the reported biological and therapeutic effects; however, more rigorous clinical trials have not established true biological activity over placebo effects [36, 37]. The scientific basis is not strong to support the claim for efficacy in application of low intensity ultrasound in medical procedures. In general, popular commercialized physiotherapy practices do show that low intensity ultrasound does not cause significant inverse effect and is relatively safe.

We have also briefly tested ultrasound of higher frequency (1 and 3 MHz) for their activity in reversing paclitaxel cytotoxicity. The initial finding was that ultrasound waves with higher frequency were also capable of breaking rigid microtubules and reducing paclitaxel-induced cell death. However, the activity of 1 and 3 MHz ultrasound seemed to be less effective than the long wavelength (45 KHz) ultrasound in the limited preliminary testings. Thus, a careful comparison of ultrasound of long wavelength to higher frequency for their ability to break paclitaxel-induced rigid microtubules and reduce cytotoxicity should be performed in the future. Likely, an optimal setting to use higher frequency ultrasound to counter paclitaxel cytotoxicity can be found, and would be useful in certain medical applications as well.

Our finding may have some implications for clinical practice. Since ultrasound disrupts the cellular cytoskeleton, it could interfere with paclitaxel efficacy as a chemotherapeutic agent. Caution in using ultrasound to image and monitor tumors following paclitaxel treatment might be warranted, though it is not investigated if the energy level used would be sufficient to break microtubule bundles. At the least, the schedule of imaging should be adjusted to minimize potentially interfering with paclitaxel activity. Still, ultrasound used in diagnostic imaging has low intensity, and the power of the ultrasound waves may not be significant to interfere with drug activity. Additionally, focused and restricted low intensity ultrasound may be applied to prevent toxicity in tissues and organs that are unintentionally exposed to paclitaxel during systemic chemotherapy. In this aspect, it may be interesting to determine if low intensity ultrasound can also reduce cytotoxicity in neuronal cells. If so, low intensity ultrasound might be used to prevent peripheral neuropathy, which is a dose limiting factor in the use of paclitaxel in cancer treatment. Similarly, if ultrasound can reverse paclitaxel cytotoxicity on the cells of hair follicles, a potential treatment for another detrimental side effect of paclitaxel chemotherapy, alopecia, may be resolved by using ultrasound.

We did not observed any killing or suppressive effects of ultrasound shock waves at the level of 1 W/cm^2^ on cells in culture. This is consistent with the low risk and widely use of low intensity ultrasound to treat various medical issues. In some experiments, a brief exposure to ultrasound was observed to increase slightly the cell growth during the following 2 days. Likely a brief exposure of cells to ultrasound stimulates cellular growth signaling pathways, such as reported stimulation of MAPK activity and cell proliferation by low intensity ultrasound [66].

We speculate that ultrasound is effective to eliminate cytotoxicity at time only when free paclitaxel in the environment become minimal. Thus, the amount of paclitaxel added and the time for the cells to sequester all paclitaxel will determine the ability of ultrasound exposure to remove paclitaxel-induced cell death and growth suppression. Paclitaxel binds to beta-tubulin with a high affinity, and in the presence of paclitaxel, nearly 80–90% of cellular tubulin becomes polymerized, whereas normally around 50% of cellular tubulin is present as a heterodimer [67]. Additionally, paclitaxel is extremely hydrophobic, and likely the molecules rapidly become associated with proteins or other cellular components. Thus, when only a low concentration of paclitaxel is added, paclitaxel likely is depleted in a short time span.

**Fig. 7:**
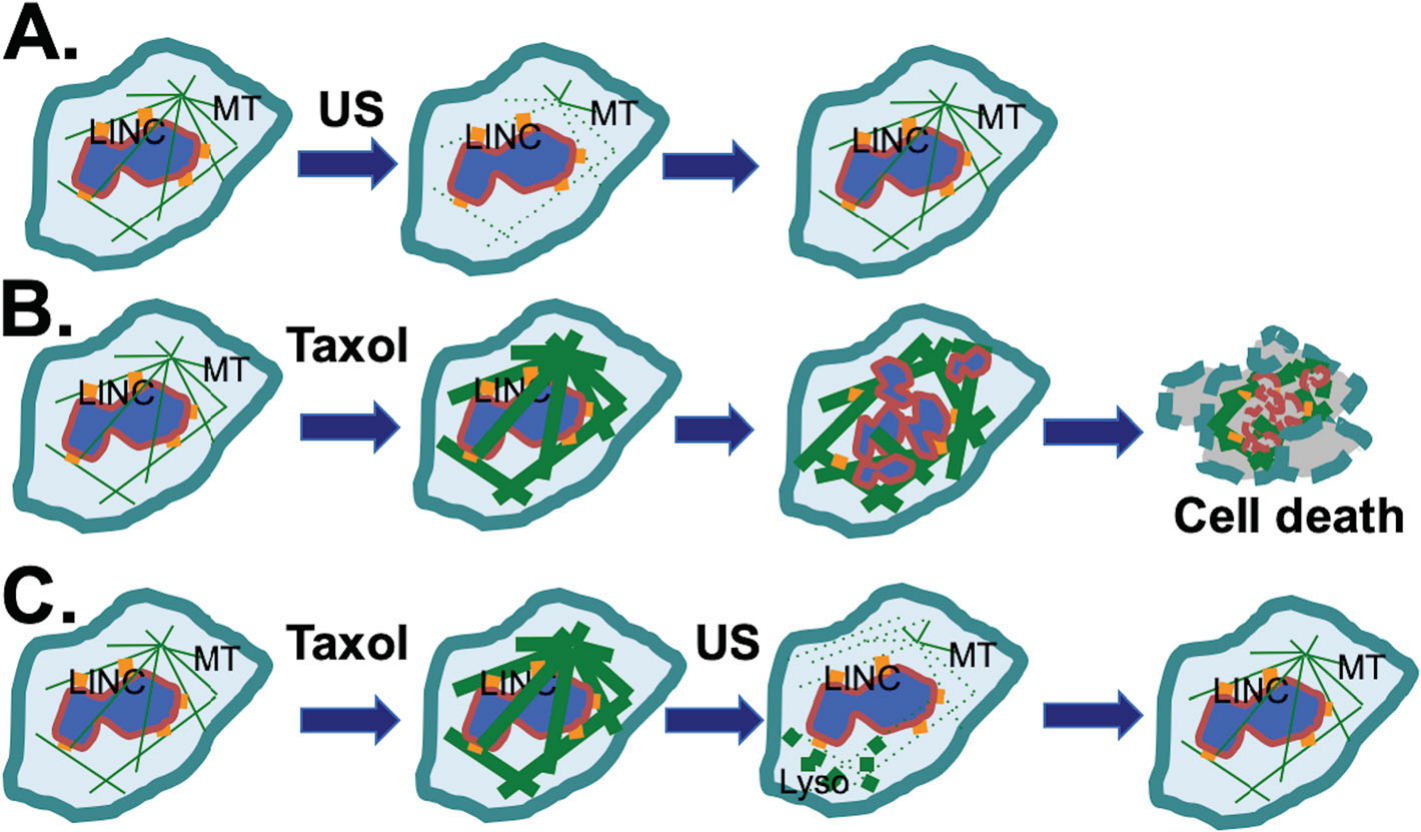
Concept: Ultrasound reverses cytotoxicity by disrupting rigid microtubule filaments induced by Taxol treatment of proliferating cells. Microtubule (MT) bundles are radiated from the organizing center and associate with the nuclear envelope through LINC (LInking Nuclear and Cytoplasmic) complexes. **A** Ultrasound (US) is known to transiently disrupt microtubule networks, which reform in a few (1, 2) h. **B** Taxol induces rigid microtubule filaments that lead to growth arrest and subsequent cell death. **C** We suggest a mechanism through which ultrasound reverses cytotoxicity by disrupting rigid microtubule filaments induced by Taxol. The Taxol-bound microtubule fragments and tubulins are relocated to lysosomes (Lyso) for degradation (illustrated by the bold dots), and tubulins without bound Taxol form a new microtubule network. Thus, a brief ultrasound exposure is able to eliminate the impact of paclitaxel on microtubules, reversing the cytotoxicity

A previous study observed that a short-term exposure of cancer cells to paclitaxel has a long-lasting inhibitory activity [65]. When associated with tubulin, paclitaxel can stabilize the microtubule filaments over 2–3 days, resulting in cell death. However, when disrupted by the physical force exerted by ultrasound shock waves, the tubulins and the bound paclitaxel undergo degradation (Fig. 7). The microtubules re-established are not associated with paclitaxel, thus ultrasound exposure eliminates the effect of paclitaxel on microtubules. Consequently, the cytotoxicity of paclitaxel on the cancer cells is lessened. A model explains this observation (Fig. 7).

Microtubules, or tubulins are stable proteins with long cellular half-life [68, 69]. Several studies report on the turn over and degradation of tubulin by cathepsin D proteasomes pathways [70, 71], but not the lysosomal pathway [71]. Our current finding that paclitaxel-bound microtubule fragments or tubulin monomers are degraded through the lysosomal pathway appears inconsistent with previous findings. However, paclitaxel-bound microtubule fragments generated following disruption by ultrasound shock waves are likely processed through a different pathway than heterodimeric tubulins. Future investigations will determine the mechanism of how ultrasound generated microtubule fragments are delivered to lysosomes.

The cellular homeostasis of tubulins is tightly regulated to ensure the presence of a constant level of tubulins for cellular function [68]. The production of alpha- and beta-tubulins is coordinated [69], and the transcription and synthesis of alpha and beta tubulin is regulated by unpolymerized tubulins [72], which participate in the transcription complex [73]. Paclitaxel may stimulate tubulin polymerization and reduces the cellular level of free tubulins, and thus increase tubulin synthesis. This newly generated tubulin would bind and sequester paclitaxel.

The current finding that a brief exposure to ultrasound reverses paclitaxel cytotoxicity by itself will not be useful for using ultrasound to enhance chemotherapy, since generally one would hope to increase cytotoxicity of the drug on cancer cells. However, the finding may caution the exposure of patients immediately after paclitaxel chemotherapy administration, which may be used for monitoring purposes in some cases. Additionally, the finding may be useful to prevent paclitaxel-caused neuronal toxicity caused by paclitaxel chemotherapy. Thus, low intensity ultrasound may be useful to prevent paclitaxel-induced peripheral neuropathy or alopecia, if the observation in cancer cells is also found to be applicable to neuronal cells or cells of hair follicles.

## Supplementary Information

**Additional file 1: Supplementary Figure 1.** Disruption of microtubule cytoskeleton in OVCAR3 ovarian cancer cells. (A) OVCAR3 ovarian cancer cells on tissue culture dish were exposed to ultrasound (+ US, 40 KHz, 3 W/cm^2^) for 5 min. The cells were then were fixed and processed for immunostaining for alpha-tubulin and Lamin A/C. (B) OVCAR3 cells on tissue culture dish were exposed to ultrasound (+ US, 40 KHz, 1 W/cm^2^) for 5 min and then were fixed and processed for analyses by immunofluorescence microscopy for alpha tubulins and Lamin B. **Supplementary Figure 2.** Disruption of fluorescence labeled Taxol bound microtubule cytoskeleton in cancer cells by ultrasound. Breast (MCF-7) and ovarian (OVCAR3, OVCAR8) cancer cells were added with fluorescence labeled Taxol analog (488-PTX) (1 nM) for 60 min. The cells on tissue culture dish were exposed to ultrasound (+ US, 40 KHz, 1.0 W/cm^2^) for 5 min. Representative 488-PTX images were taken before (control) and after exposure to ultrasound. **Supplementary Figure 3.** Disruption and progressively elimination of fluorescence labeled Taxol bound microtubule cytoskeleton in ovarian cancer cells by ultrasound. OVCAR3 ovarian cancer cells were added with fluorescence labeled Taxol analog (488-PTX) (1 nM) for 60 min. The cells on tissue culture dish were exposed to ultrasound (+ US, 40 KHz, 1.0 W/cm^2^) for 5 min. Representative 488-PTX images were taken before (control) and after exposure to ultrasound for 0, 10, 20, and 30 min.

## Data Availability

Cell lines (some are available commercially from ATCC), more detailed lab procedures and protocols, and the datasets used and/or analyzed during the current study are available from the corresponding author on reasonable request.
