# Supplementary material for: Exposure to low intensity ultrasound removes paclitaxel cytotoxicity in breast and ovarian cancer cells
Source: BMC Cancer. 2021 Sep 1;21:981. doi: 10.1186/s12885-021-08722-7 (PMC8408969; doi:10.1186/s12885-021-08722-7)

Supplementary Figure 1

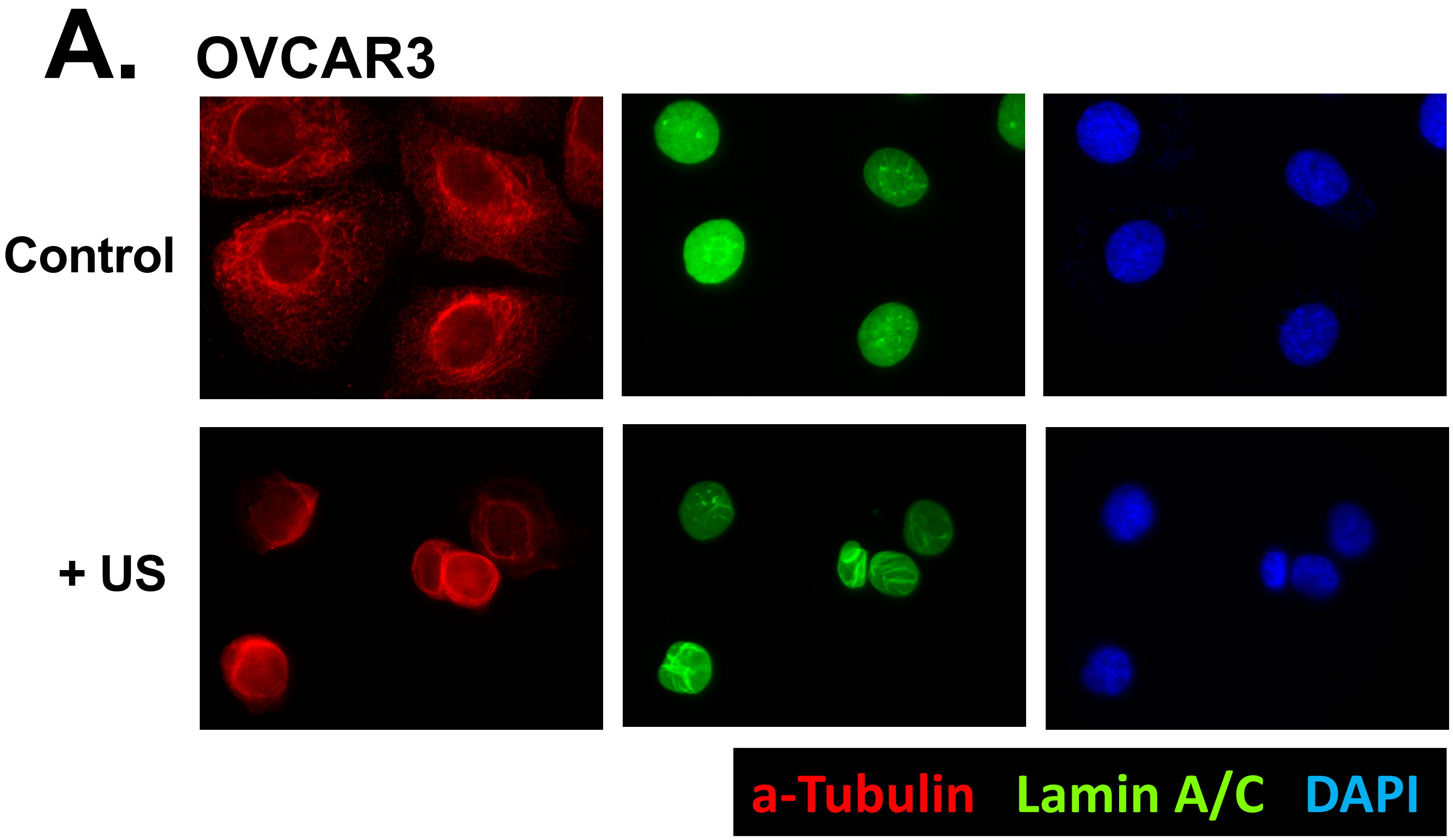

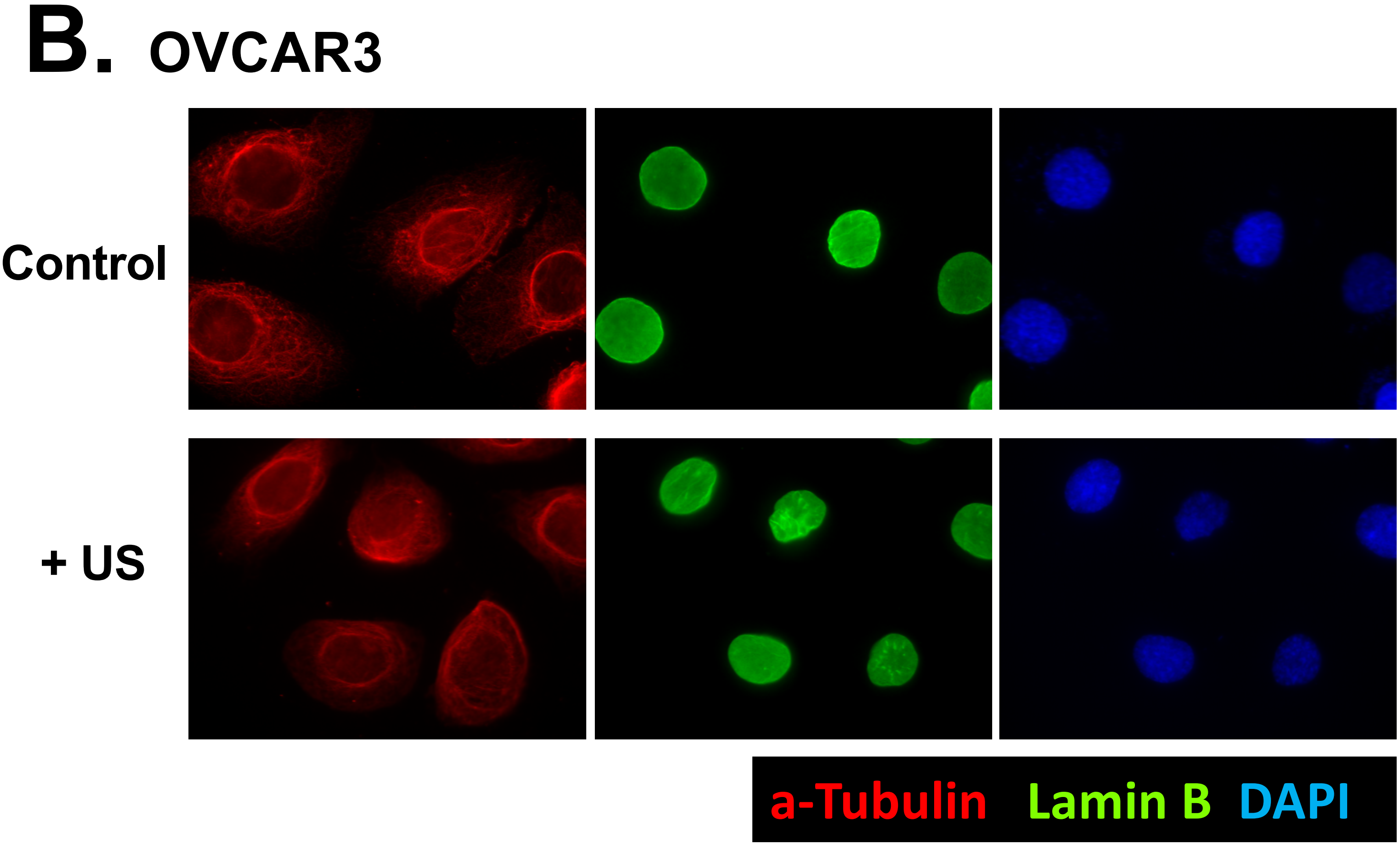

Supplementary Figure 2

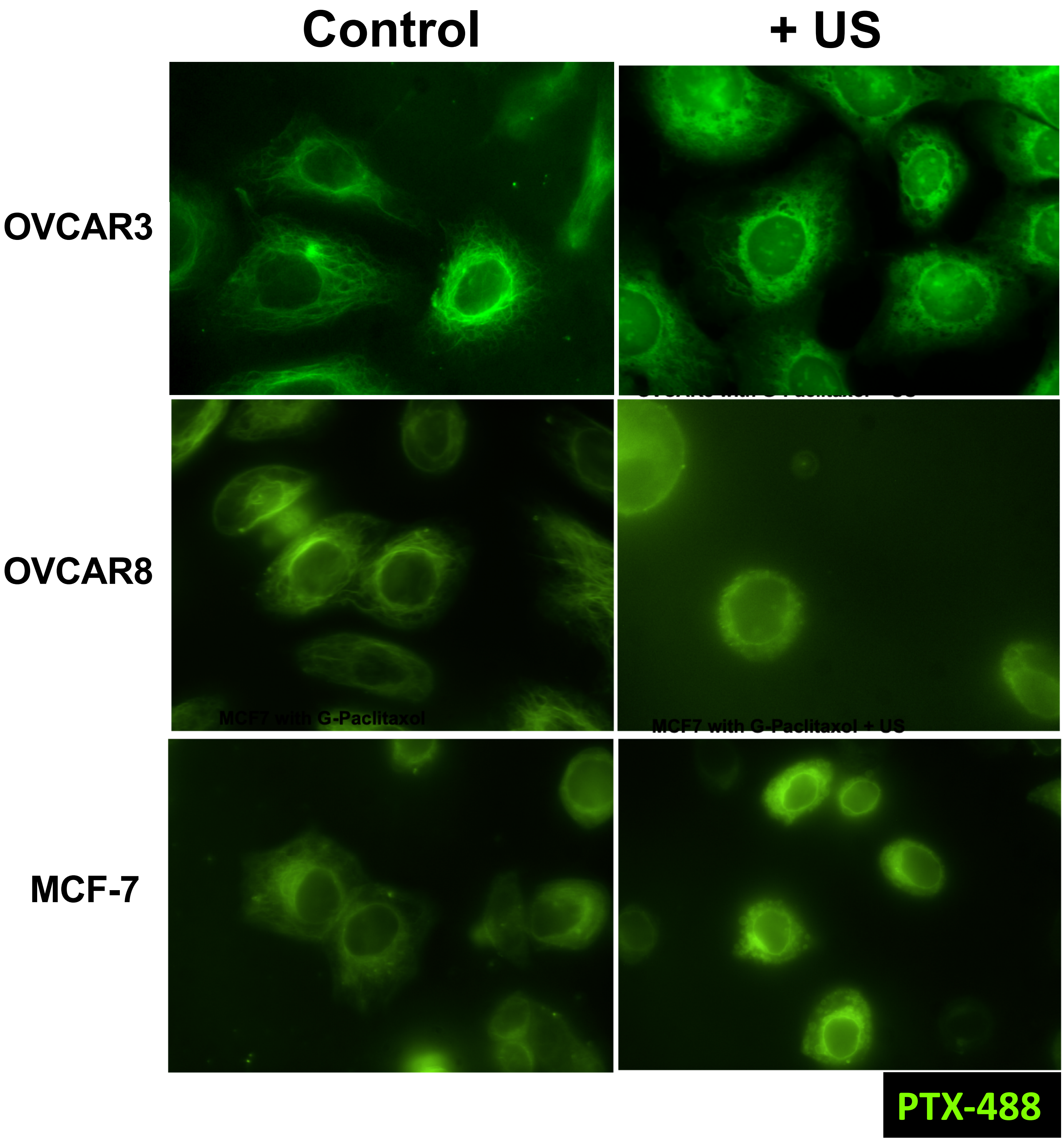

Supplementary Figure 3

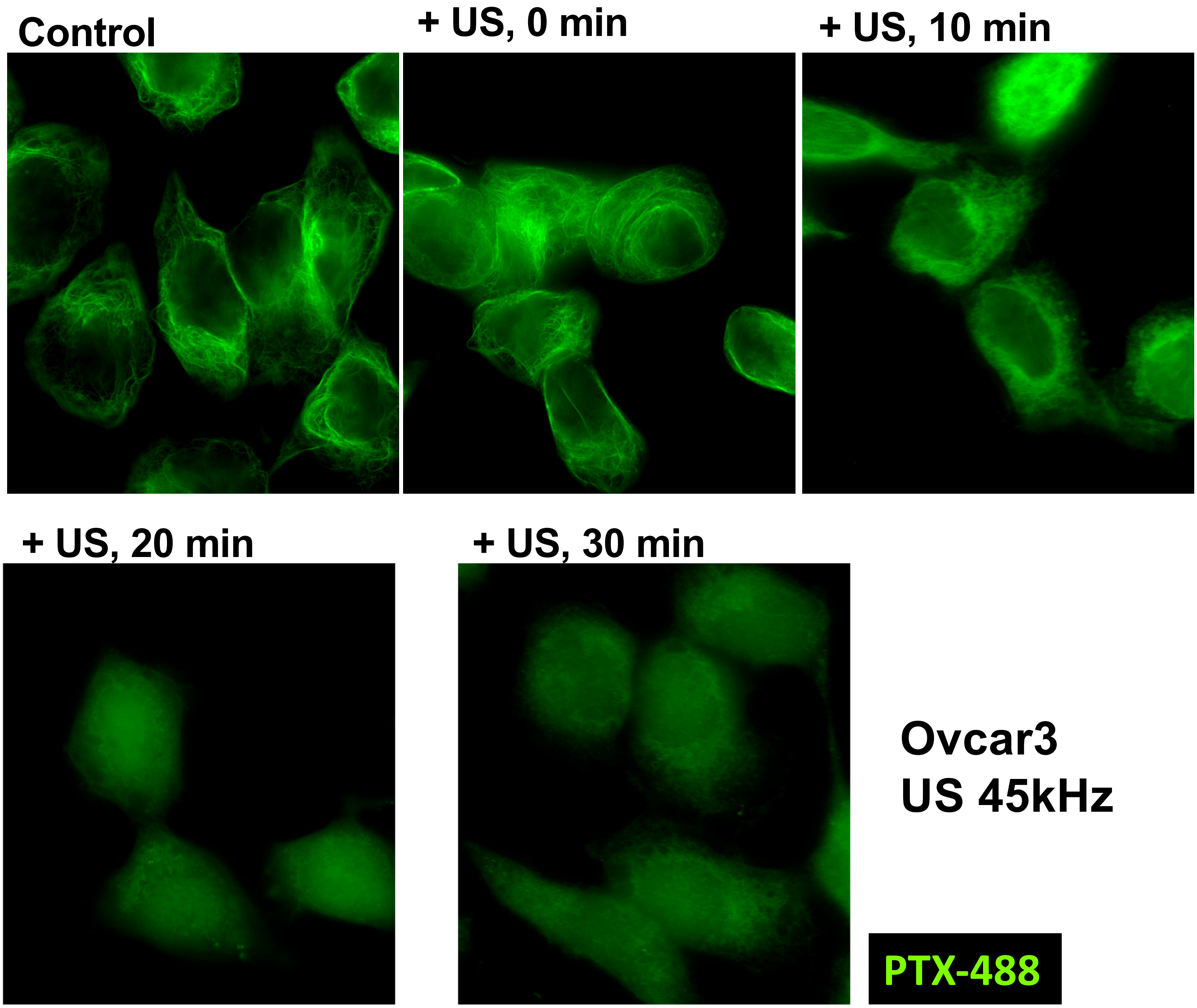

Supplement: Supplementary file 1 — Additional file 1: Supplementary Figure 1. Disruption of microtubule cytoskeleton in OVCAR3 ovarian cancer cells. (A) OVCAR3 ovarian cancer cells on tissue culture dish were exposed to ultrasound (+ US, 40 KHz, 3 W/cm2) for 5 min. The cells were then were fixed and processed for immunostaining for alpha-tubulin and Lamin A/C. (B) OVCAR3 cells on tissue culture dish were exposed to ultrasound (+ US, 40 KHz, 1 W/cm2) for 5 min and then were fixed and processed for analyses by immunofluorescence microscopy for alpha tubulins and Lamin B. Supplementary Figure 2. Disruption of fluorescence labeled Taxol bound microtubule cytoskeleton in cancer cells by ultrasound. Breast (MCF-7) and ovarian (OVCAR3, OVCAR8) cancer cells were added with fluorescence labeled Taxol analog (488-PTX) (1 nM) for 60 min. The cells on tissue culture dish were exposed to ultrasound (+ US, 40 KHz, 1.0 W/cm2) for 5 min. Representative 488-PTX images were taken before (control) and after exposure to ultrasound. Supplementary Figure 3. Disruption and progressively elimination of fluorescence labeled Taxol bound microtubule cytoskeleton in ovarian cancer cells by ultrasound. OVCAR3 ovarian cancer cells were added with fluorescence labeled Taxol analog (488-PTX) (1 nM) for 60 min. The cells on tissue culture dish were exposed to ultrasound (+ US, 40 KHz, 1.0 W/cm2) for 5 min. Representative 488-PTX images were taken before (control) and after exposure to ultrasound for 0, 10, 20, and 30 min. [file 12885_2021_8722_MOESM1_ESM.pdf]
